# Fish as Hosts of *Vibrio cholerae*

**DOI:** 10.3389/fmicb.2017.00282

**Published:** 2017-02-28

**Authors:** Malka Halpern, Ido Izhaki

**Affiliations:** ^1^Department of Biology and Environment, Faculty of Natural Sciences, University of HaifaTivon, Israel; ^2^Department of Evolutionary and Environmental Biology, Faculty of Natural Sciences, University of HaifaHaifa, Israel

**Keywords:** fish, *Vibrio cholerae*, waterbird, bacteria–fish interactions, reservoir, vector

## Abstract

*Vibrio cholerae*, the causative agent of pandemic cholera, is abundant in marine and freshwater environments. Copepods and chironomids are natural reservoirs of this species. However, the ways *V. cholerae* is globally disseminated are as yet unknown. Here we review the scientific literature that provides evidence for the possibility that some fish species may be reservoirs and vectors of *V. cholerae*. So far, *V. cholerae* has been isolated from 30 fish species (22 freshwater; 9 marine). *V. cholerae* O1 was reported in a few cases. In most cases *V. cholerae* was isolated from fish intestines, but it has also been detected in gills, skin, kidney, liver and brain tissue. In most cases the fish were healthy but in some, they were diseased. Nevertheless, Koch postulates were not applied to prove that *V. cholerae* and not another agent was the cause of the disease in the fish. Evidence from the literature correlates raw fish consumption or fish handling to a few cholera cases or cholera epidemics. Thus, we can conclude that *V. cholerae* inhabits some marine and freshwater fish species. It is possible that fish may protect the bacteria in unfavorable habitats while the bacteria may assist the fish to digest its food. Also, fish may disseminate the bacteria in the aquatic environment and may transfer it to waterbirds that consume them. Thus, fish are reservoirs of *V. cholerae* and may play a role in its global dissemination.

## Introduction

The devastating disease, cholera, is known to occur globally causing epidemics and pandemics. However, the way this disease is worldwide disseminated is still unknown. *Vibrio cholerae*, the causing agent of cholera is ubiquitous in marine and freshwater aquatic environments. Copepods (*Crustacean*) (Colwell and Huq, 2001) and chironomids (*Diptera; Chironomidae*) (Broza and Halpern, 2001; Halpern et al., 2004, 2006, 2007; Senderovich et al., 2008; Halpern and Senderovich, 2015) were described as natural reservoirs of *V. cholerae*. Copepods and chironomids are abundant in fresh and marine water ecosystems and are consumed by different fish species. Halpern et al. (2008) raised the hypothesis that fish that feed on copepods and chironomids, and waterbirds that also may feed on these invertebrates and consume fish as well, may be reservoirs and vectors of *V. cholerae*. Here we review the scientific literature that indicates that fish are indeed significant reservoirs of *V. cholerae* in water ecosystems.

### Vibrio cholerae

*V. cholerae*, a Gram-negative motile rod causes massive cholera outbreaks such as the one following the 2010 earthquake in Haiti (Sack et al., 2004; Chin et al., 2011; Katz et al., 2013). Cholera is a global threat to public health and it was estimated that between 2008 and 2012 cholera caused an annual average of 2.9 million cases, and 95,000 deaths, worldwide (Ali et al., 2015). Particular serogroups (O1 and O139) of this bacterium are responsible for cholera epidemics and pandemics. Human infection with *V. cholerae* begins with ingestion of contaminated food or water containing the bacterium. *V. cholerae* colonizes the small intestine and secretes cholera enterotoxin (CT) into the host cells resulting in rapid efflux of chloride ions and water into the lumen of the intestine, leading to profuse diarrhea and severe dehydration (Kaper et al., 1995).

Non-O1/non-O139 *V. cholerae* serogroups are also linked to *V. cholerae* gastroenteritis as well as to wound infections and bacteremia (Deshayes et al., 2015). *V. cholerae* O1, O139 and non-O1/O139 comprise a single taxonomic species and their habitats attributes are similar (Lewin, 1996), however, recently it has been suggested that not all strains of *V. cholerae* species share the same niche (Kirchberger et al., 2016). The role of CT in the environment is not understood.

*V. cholerae* is commonly associated with chitin-containing zooplankton, particularly copepods (Huq et al., 1983) and chironomids (Broza and Halpern, 2001; Halpern et al., 2004). Recent evidence supports the hypothesis that fish and waterbirds may also be intermediate reservoirs and vectors of *V. cholerae* (Halpern et al., 2008; Halpern and Izhaki, 2010).

### Fish as possible reservoirs of *V. cholerae*

#### *V. cholerae* O1 and O139 serogroups in fish

In a laboratory experiment that was conducted more than 50 years ago, Felsenfeld (1963), infected sardines (*Stolephorus*) and mullets (*Liza*) with pathogenic *V. cholerae* O1 strains (Ogawa and Inaba). *Vibrio* concentration in the water was 10^2^ cells/ml. The strains were detected in the fish intestine after the fish were exposed to the bacteria (Table 1). In another laboratory experiment, Runft et al. (2014) used *V. cholerae* O1 strains to colonize zebrafish gut. They found that the bacteria attached to the fish intestinal epithelium and formed micro-colonies. They suggested that zebrafish can act as a host model for pathogenic *V. cholerae* strains (Rowe et al., 2014; Runft et al., 2014) (Table 1). Evidence for the presence of pathogenic serogroups of *V. cholerae* in fish was published by du Preez et al. (2010) who detected large numbers of *V. cholerae* O1 and O139 in fish scale samples collected in Mozambique. These researchers obtained their evidence by a direct fluorescent antibody technique. *V. cholerae* O1, positive for cholera toxin gene, was isolated from Tilapia gills in Tanzania (Hounmanou, 2015). *V. cholerae* O1 isolates, positive to *ctxA* and *tcpA* genes were detected from two marine fish in Cochin, India (no details were given as to the fish species) (Kumar and Lalitha, 2013). In the same study, Kumar and Lalitha (2013) also identified 141 non-O1/O139 isolates from unidentified marine fish species (Table 1).

**Table 1:** **Isolation of *V. cholerae* strains from healthy fish species that were sampled from different habitats and regions around the world**.

**Fish species**	**Habitat**	**Site of isolation**	**Isolated from fish organ/comments**	**References**
***V. cholerae*** **O1**				
Sardines (*Stolephorus* sp.)	Intestine colonization lab experiment	Medical Research Laboratory, Bangkok, Thailand	Intestine, survival in the intestine lasted only 5 days	Felsenfeld, 1963
Mullet (*Liza* sp.)	Intestine colonization lab experiment	Medical Research Laboratory, Bangkok, Thailand	Intestine, O1 survival in the intestine lasted only 5 days	Felsenfeld, 1963
Unidentified sea fish,	Beira beach	The Pungwe estuary at Beira, Mozambique	*V. cholerae* O1/O139 were detected on fish scale using direct fluorescent antibody	du Preez et al., 2010
Unidentified sea fish	Marin environment	Cochin, India	*V. cholerae* O1, and non O1	Kumar and Lalitha, 2013
Zebrafish (*Danio rerio*)	Intestine colonization lab experiment (demonstrating a host model)	Wayne State University IACUC, Michigan, USA	O1, Intestine, micro-colonies were observed on the intestinal epithelium	Runft et al., 2014
*Tilapia* sp.	Mzumbe sewage stabilization ponds	Morogoro, Tanzania	*V. cholerae* O1, and non-O1 from gills *V. cholerae* non-O1 from intestine samples	Hounmanou, 2015
***V. cholerae*** **NON O1/O139**				
lorna fish (*Sciaena deliciosa*)	Marine inshore waters	Peru	Information not available	Carvajal et al., 1988
*Tilapia* sp.	Fish pond	Haifa and Nir David, Israel	Intestine	Halpern et al., 2008
Common St. Peter's fish (*Tilapia* sp. and *Tilapia zillii*)	Fish pond	Nahalal, Israel	Intestine	Senderovich et al., 2010
Josephus cichlid (*Astatotilapia flaviijosephi*)	Fish pond	Nir David, Israel	Intestine	Senderovich et al., 2010
Grass carp, white-amur (*Ctenopharyngodon idella*)	Fish pond	Atlit, Israel	Intestine	Senderovich et al., 2010
Common carp (*Cyprinus carpio*)	Fish pond	Atlit, Israel	Intestine	Senderovich et al., 2010
Flathead gray mullet (*Mugil cephalus*)	Fish pond	Nahalal, Israel	Intestine	Senderovich et al., 2010
Galilee St. Peter's fish (*Sarotherodon galilaeus*)	Fish pond	Kfar Rupin, Israel	Intestine	Senderovich et al., 2010
Jordan St. Peter's fish (*Oreochromis aureus*)	River	Nir David, Israel	Intestine	Senderovich et al., 2010
*Carasobarbus canis*	Lake	The Sea of Galilee, Israel	Intestine	Senderovich et al., 2010
Longhead barbel (*Barbus longiceps*)	Lake	The Sea of Galilee, Israel	Intestine	Senderovich et al., 2010
Flathead gray mullet (*Mugil cephalus*)	Lake	The Sea of Galilee, Israel	Intestine	Senderovich et al., 2010
Blotcheye soldierfish (*Myripristis murdjan*)	Mediterranean Sea (Marine water)	Akko, Israel	intestine	Senderovich et al., 2010
Bulls eye (*Priacanthus hamrur*)	Royapuram coast (Marine water)	Chennai, Tamil Nadu, India	Intestine and the muscles	Sujatha et al., 2011
Hard tail scad (*Megalaspis cordyla*)	Royapuram coast (Marine water)	Chennai, Tamil Nadu, India	Gills, intestine, muscles and skin	Sujatha et al., 2011
Zebrafish (*Danio rerio*)	Adult zebrafish cultured in tanks	Auckland, New Zealand	*V. cholerae* was detected in intestine samples using cloning of 16S rRNA gene	Lan and Love, 2012
Turbot fish (*Scophthalmus maximus*)	Marine aquaculture	Qingdao, China	*V. cholerae* was detected by metagenomic tools	Xing et al., 2013
Sheepshead (*Archosargus probatocephalus*)	Fowl River (estuarine)	Gulf of Mexico	Intestine	Jones et al., 2013
Sea catfish (*Arius felis*)	Fowl River (estuarine)	Gulf of Mexico	Intestine	Jones et al., 2013
Pin fish (*Lagodon rhomboides*)	Fowl River (estuarine)	Gulf of Mexico	Intestine	Jones et al., 2013
Crevalle jack (*Caranx hippos*)	Fowl River (estuarine)	Gulf of Mexico	Intestine	Jones et al., 2013
Frozen tra fish (*Pangasius hypophthalmus*) fillet	Food industry	Vietnam	Final packaged products (fillets)	Thi et al., 2014
Tilapia (*Oreochromis niloticus*)	Tanghin freshwater reservoir	Ouagadougou, Burkina Faso (Africa)	6.3% (15 out of 238)	Traoré et al., 2014
**INDIRECT INDICATION FOR** ***V. cholerae*** **PRESENCE IN FISH**				
Unknown tropical fish	Water from Fish tank	UK	Reported to be the cause of a wound	Booth et al., 1990
Common goldfish (*Carassius auratus*)	Aquarium water	Rhode Island	Indication using molecular methods	Smith et al., 2012
***V. cholerae*** **ISOLATED FROM DISEASED FISH**				
Ayu fish (*Plecoglossus altivelis*) and Guppy Fish (*Poecilia reticulate*)	River	Japan	Livers, spleens, or kidneys of diseased fish	Yamanoi et al., 1980; Kiiyukia et al., 1992
Goldfish (*Carassius auratus*)	No data available	No data available	No data available	Reddacliff et al., 1993
Nile tilapia (*Oreochromis niloticus*)	Floating cage cultured Nile tilapia farms	Mekong River, Thailand	*V. cholerae* from internal organs of diseased fish	Dong et al., 2015
Guppy Fish (*Poecilia reticulate*)	Aquaculture ponds	Kasha, Iran	Skin, gill, kidney and brain tissue from diseased fish	Kiani et al., 2016
Cardinal tetra (*Paracheirodon axelrodi*)	Fish aquarium	Czech Republic	Diseased fish	Rehulka et al., 2015
Raphael catfish (*Platydoras costatus*)	Fish aquarium	Czech Republic	Diseased fish	Rehulka et al., 2015
Common nase (*Chondrostoma nasus*)	Fish aquarium	Czech Republic	Diseased fish	Rehulka et al., 2015

#### *V. cholerae* non-O1/O139 in fish

Carvajal et al. (1988) identified *V. cholerae* non-O1/O139 serogroups in healthy Lorna fish (*Sciaena deliciosa*) sampled from inshore marine sites during a Peruvian cholera epidemic (Table 1). Senderovich et al. (2010) examined freshwater and marine fish species. Ten freshwater (71%) and one marine (2.3%) fish species tested positive for the presence of *V. cholerae* non-O1/O139 in their intestine (Table 1). *V. cholerae* non-O1/O139 was also detected in four fish species collected from the Fowl River in the Gulf of Mexico (Jones et al., 2013) (Table 1). The prevalence of *V. cholerae* isolates in Tilapia (*Oreochromis niloticus*) intestines, sampled from a water reservoir in Ouagadougou, Burkina Faso in Africa, was 6.3% (Traoré et al., 2014) (Table 1). In Qingdao in China, *V. cholerae* was detected by means of metagenomic tools in the gastrointestinal tract of a farmed adult turbot fish (*Scophthalmus maximus*) (Xing et al., 2013). In India, *V. cholerae* was isolated from two fish species (Bulls eye, *Priacanthus hamrur* and Hard tail scad, *Megalaspis cordyla*) caught off Royapuram coast (Sujatha et al., 2011) (Table 1). When the microbial quality and safety of *Pangasius* fish processed for export in Vietnam was evaluated, *V. cholerae* was isolated from tra fish (*Pangasius hypophthalmus*) fillets and from the water used to rinse them (Thi et al., 2014) (Table 1).

#### *V. cholerae* isolated from diseased fish

A few studies have reported the isolation of *V. cholerae* non-O1/O139 from diseased fish. *V. cholerae* was isolated from internal organs of diseased ayu (*Plecoglossus altivelis*) and guppy fish (*Poecilia reticulate*) in Japan and Iran, respectively (Yamanoi et al., 1980; Kiiyukia et al., 1992; Kiani et al., 2016) and from Nile tilapia (*Oreochromis niloticus*) that were cultured in floating cages in Thailand (Dong et al., 2015) (Table 1). Rehulka et al. (2015) demonstrated that an intraperitoneal injection of *V. cholerae* into common carp, rainbow trout and common nase caused the death of the injected fish (Table 1).

#### Indirect evidence on the possible presence of *V. cholerae* in fish

According to the Hong Kong Food and Environmental Hygiene Department, *V. cholerae* serotype Ogawa biotype El Tor was found in a supermarket fish tank water in Pok Fu Lam in September 2003 (Press Release, 2003). They were not able to explain the source of the bacteria. *V. cholerae* O1 was detected from aquarium water and fish imported from Thailand and Sri Lanka to Czech Republic (Plesník and Procházková, 2006). Using molecular methods, Smith et al. (2012), identified *V. cholerae* from aquarium water containing common goldfish (*Carassius auratus*) purchased from aquarium shops in Rhode Island (Table 1). When the bacterial community of zebrafish intestinal tracts was studied using cloning of the 16S rRNA gene, *V. cholerae* was found as the dominant OUT in the *Gammaproteobateria* class (Lan and Love, 2012).

### Epidemiological evidence of fish consumption as the cause of cholera

Evidence from the literature correlated fish with a few cholera cases or epidemics. The first records date back over more than 50 years. It was postulated that cholera endemicity in India was due to hilsa fish (Pandit and Hora, 1951) (Table 2). Morgan et al. (1960), suggested that the origin of El Tor *vibrios* outbreaks in Thailand might have been fish that are often eaten raw in the Pacific area. Cholera was associated with eating salted fish, sardines and other fish from an atoll lagoon in the Pacific Ocean (Merson et al., 1977; Kuberski et al., 1979; McIntyre et al., 1979). A cholera outbreak in Tanzania (67 patients, including 11 deaths) was correlated with handling and eating fish at social gatherings (Killewo et al., 1989). Out of 12 cholera cases caused by *V. cholerae* O1, serotype Ogawa, biotype El Tor, in the southern Italian region of Puglia, in 1994, three patients reported consumption of raw fish (Maggi et al., 1997) (Table 2). Consumption of dried fish correlated significantly with cholera risk in Tanzania (Acosta et al., 2001). In July 2001, a case of cholera, caused by *V. cholerae* O1, serovar Inaba, biovar El Tor, was reported in Berlin. Interestingly, the patient had most likely been infected while handling and preparing fish imported from Nigeria (Schürmann et al., 2002). A food trace-back investigation following three cases of cholera in Sydney, Australia, found that the only exposure common to all cases was consumption of raw whitebait imported from Indonesia (Forssman et al., 2007). *V. cholerae* O1 serovar Ogawa was identified as the causative agent in all three cases. *V. cholerae* non-O1 was isolated from stools of a fisherman who had fished, cooked and eaten a lake fish in Italy (Piantieri et al., 1982). The source of *Vibrio cholerae* non-O1 that was found in a wound, was linked with a tropical fish tank (Booth et al., 1990) (Table 2).

**Table 2:** **Fish consumption as the source of cholera disease**.

**Etiology**	**Fish source**	**Site**	**Serogroup**	**References**
Hilsa fish (*Hilsa ilisa*) was connected to the transmission of endemic cholera. *Hilsa* fish, infected with *V. cholerae* breeds abundantly in the Hoogly river that runs through Calcutta	India	Cholera endemicity in India due to the Hilsa fish	*V. cholerae* O1	Pandit and Hora, 1951
Eating of raw fish	Thailand	Outbreaks in Thailand, 1959	*V. cholerae* O1El Tor	Morgan et al., 1960
Correlated with handling and eating fish at social gatherings	Tanzania	Cholera outbreak in Tanzania	*V. cholerae* O1	Killewo et al., 1989
Fishing, cooking and eating a lake fish	A lake in Italy	Italy	*V. cholera* non O1	Piantieri et al., 1982
Eating of raw fresh and smoked fish	Guinea	Conakry, Guinea	*V. cholerae* O1	St. Louis et al., 1990
Eating of small salted fish	The Pacific Ocean	Island of Guam, 1974		Merson et al., 1977
Salted fish	The Pacific Ocean	Gilbert Island 1977		McIntyre et al., 1979; Kuberski et al., 1979
A cholera outbreak in (67 patients, including 11 deaths) was correlated with handling and eating fish at social gatherings	Tanzania	Butiama village of the Mara Region, Tanzania	*V. cholerae* O1	Killewo et al., 1989
Consumption of raw fish illegally imported from Albania	Unknown fish species, imported from Albania to Italy	Italy, 1994	*V. cholerae* O1	Maggi et al., 1997
Eating of dried fish		Rural area (Ifakara) in southern Tanzania, Africa	*Vibrio cholerae* O1**, biotype El Tor, serotype Ogawa	Acosta et al., 2001
A patient contracted the infection while handling a fish imported from Nigeria	Nigeria	Germany, 2001	*V. cholerae* O1 serovar Inaba, biovar El Tor	Schürmann et al., 2002
Eating of whitebait imported from Indonesia	Indonesia	Sydney Australia, 2006	*Vibrio cholerae* O1 biotype El Tor, serotype Ogawa	Forssman et al., 2007

### *V. cholerae* and fish—mutualistic interactions?

A few publications (mentioned above and in Table 1), correlated the presence of *V. cholerae* with a disease in fish (Yamanoi et al., 1980; Kiiyukia et al., 1992; Dong et al., 2015; Rehulka et al., 2015; Kiani et al., 2016). Kiiyukia et al. (1992), who isolated *V. cholerae* non-O1/O139 from diseased ayu fish in Japan, emphasized that healthy ayu fish caught in Lake Biwa, Japan in the rivers running into this lake, also harbored *V. cholerae* but without showing any signs of a disease. Kiani et al. (2016) isolated *V. cholerae* along with other pathogens from diseased Nile tilapia that were cultured in floating cages in Thailand. However, it was not proven that *V. cholerae* was indeed the causative agent of the disease (Table 1). All the above studies simply assumed that because they isolated *V. cholerae* from the diseased fish, this species was responsible for the disease. Rehulka et al. (2015) injected a fish with relatively large dose of bacteria (e.g., 2 × 10^8^ cells) to obtain fish mortality but without following all Koch postulates rules. Hence we argue that at least for some cases other bacterial species or viruses and not *V. cholerae* were probably responsible for the fish disease (Table 1).

Senderovich et al. (2010) isolated *V. cholerae* from 15 different heathy fish species. They found 5 × 10^3^ and 1.4 × 10^2^ colony forming units (cfu) of *V. cholerae* per gr intestine content in *Sarotherodon galilaeus* (Galilee St. Peter's fish) and in *Mugil cephalus* (Flathead gray mullet), respectively. None of these fish showed any signs of disease. Nevertheless, there is a scarcity of quantitative studies of *V. cholerae* in fish. Many other studies reported the presence of *V. cholerae* in different healthy fish species that were sampled from both marine and freshwater habitats (listed in Table 1) but these studies did not quantify the numbers of *V. cholerae* in the fish.

Not all the fish species are inhabited by *V. cholerae*. For example, Jones et al. (2013) detected *V. cholerae* only in 4 out of 10 fish species sampled in the Gulf of Mexico (estuarine habitat). Similarly, Senderovich et al. (2010) did not detect *V. cholerae* in 4 out of 14 freshwater and in 43 out of 44 marine fish species. Scrutiny of the list of the fish species found to host *V. cholerae* revealed that all belonged to *Actinopterygii* class (Table S1). *V. cholerae* was identified from 30 species belonging to 9 different orders within this class.

Fish may actually benefit from *V. cholerae* that inhabit their intestine. Strains of *V. cholerae* secrete extracellular enzymes such as proteases (Halpern et al., 2003) and chitinases (Pruzzo et al., 2008; Senderovich et al., 2010). These enzymes may have a role in the digestion of macromolecules like proteins and chitin in the fish gut. Chitin, a polymer of β 1,4 N-acetylglucoseamine, is the main component of crustaceans' (copepods) and insects' (chironomids) exoskeletons. This insoluble polymer is a source of carbon and nitrogen (Cohen-Kupiec and Chet, 1998; Laviad et al., 2016). Senderovich et al. (2010) found that all *V. cholerae* strains isolated from 15 different fish species were able to degrade chitin. Thus, it is possible that the fish intestine serves as hosts for *V. cholerae* while the bacteria may play a role in helping the fish digest its chitinous zooplankton prey. As the fish that carry the bacteria swim from one location to another (some fish species move from rivers to lakes or sea and the reverse), they serve as vectors for *V. cholerae*. Nevertheless, fish are consumed by waterfowls, which disseminate the bacteria on a global scale (Halpern et al., 2008; Halpern and Izhaki, 2010).

## Concluding remarks

*V. cholerae* non-O1 as well as O1 and O139 inhabit highly diverse fish species. In most cases it seems that the bacteria cause the fish no harm; on the contrary, *V. cholerae* may be a part of the normal flora of at least some of the fish species, like tilapia and carp. Fish might have a mutualistic relationship with *V. cholerae*. The fish provide food and shelter for this bacterium while the bacterium may assist the fish to digest its food (e.g., chitin and protein). From an epidemiological point of view, the fish carry the cholera bacteria from one place to another. So eventually, if waterbirds feed on the fish, *V. cholerae* may be transferred in some waterbird species' digestive tracts and thus be globally spread.

## Unresolved questions and future research

1. Copepods and chironomids are natural reservoirs of *V. cholerae*. Do fish that feed on these zooplankton species get infected with *V. cholerae*?
2. Is *V. cholerae* transferred vertically or horizontally among fishes? Does an infected female transfer *V. cholerae* to her offspring?
3. Can the bacteria be transferred from one fish species' droppings to another fish species that lives in the same habitat?
4. When the fish intestine becomes infected with *V. cholerae*, does the bacteria become part of its normal microbiota?
5. What are the differences between fish species that carry *V. cholerae* in fresh and marine waters?
6. Does *V. cholerae* prevalence in fish vary by season? Or by different fish age and gender?
7. Can we determine a model fish species that carries *V. cholerae* as against those fish species that do not?

## Author contributions

MH and II wrote the manuscript and contributed the funding support.

## Funding

This work was supported in part by a grant from the Israel Science Foundation (ISF grant no. 1094/12) and in part by the Binational Science foundation (BSF grant no. 2015103).

### Conflict of interest statement

The authors declare that the research was conducted in the absence of any commercial or financial relationships that could be construed as a potential conflict of interest.
