# Supplementary material for: Fish as Hosts of Vibrio cholerae
Source: Front Microbiol. 2017 Feb 28;8:282. doi: 10.3389/fmicb.2017.00282 (PMC5328977; doi:10.3389/fmicb.2017.00282)
Supplement: Supplementary file 1 [file Table1.DOCX]

**Table S1. A list of the fish species from which *V. cholerae* was identified.** The list specifies the phylogenetic position of each species. All fish species belonged to the class *Actinopterygii* in the phylum *Chordata*.

| **Order** | **Family** | **Genus** | **Species** |
| --- | --- | --- | --- |
| [*Perciformes*](https://en.wikipedia.org/wiki/Perciformes) |  |  |  |
|  | [*Sciaenidae*](https://en.wikipedia.org/wiki/Sciaenidae) |  |  |
|  |  | *Sciaena* | *S. deliciosa* (Lorna fish) |
|  | [*Cichlidae*](https://en.wikipedia.org/wiki/Cichlidae) |  |  |
|  |  | *Tilapia* | *T. zillii* (Common St. Peter's fish) |
|  |  | [*Astatotilapia*](https://en.wikipedia.org/wiki/Astatotilapia) | *A. flaviijosephi* (Josephus cichlid) |
|  |  | *Oreochromis* | *O. aureus* (Jordan St. Peter's fish) |
|  |  |  | *O. niloticus* |
|  |  | [*Sarotherodon*](https://en.wikipedia.org/wiki/Sarotherodon) | *S. galilaeus* (Galilee st. Peter's fish) |
|  | [*Priacanthidae*](https://en.wikipedia.org/wiki/Priacanthidae) |  |  |
|  |  | [*Priacanthus*](https://en.wikipedia.org/wiki/Priacanthus) | *P. hamrur* (Bulls eye) |
|  | [*Carangidae*](https://en.wikipedia.org/wiki/Carangidae) |  |  |
|  |  | *Megalaspis* | *M. cordyla* (Hard tail scad) |
|  |  | *Caranx* | *C. hippos* (Crevalle jack) |
|  | [*Sparidae*](https://en.wikipedia.org/wiki/Sparidae) |  |  |
|  |  | [*Archosargus*](https://en.wikipedia.org/wiki/Archosargus) | *A. probatocephalus* (Sheepshead) |
|  | [*Percoidea*](https://en.wikipedia.org/wiki/Percoidea) |  |  |
|  |  | *Megalaspis* | *M. cordyla (Hard tail scad)* |
|  |  | *Lagodon* | *L. rhomboids* (pinfish) |
| [*Cypriniformes*](https://en.wikipedia.org/wiki/Cypriniformes) |  |  |  |
|  | [*Cyprinidae*](https://en.wikipedia.org/wiki/Cyprinidae) |  |  |
|  |  | *Barbus* | *B.* *longiceps* (Longhead barbel) |
|  |  | [*Carassius*](https://en.wikipedia.org/wiki/Carassius) | *Ca. auratus* (Common goldfish) |
|  |  | [*Carasobarbus*](https://en.wikipedia.org/wiki/Carasobarbus) | *Carasobarbus canis* |
|  |  | *Cyprinus* | *Cy. Carpio* (Common carp) |
|  |  | [*Chondrostoma*](https://en.wikipedia.org/wiki/Chondrostoma) | *Ch. nasus* |
|  |  | *Ctenopharyngod* | *Ct. idella* (Grass carp, white-amur) |
|  |  | [*Danio*](https://en.wikipedia.org/wiki/Danio) | *D. rerio* (Zebrafish) |
|  | [*Poeciliidae*](https://en.wikipedia.org/wiki/Poeciliidae) |  |  |
|  |  | [*Poecilia*](https://en.wikipedia.org/wiki/Poecilia) | *P. reticulate* (Guppy Fish) |
| [*Mugiliformes*](https://en.wikipedia.org/wiki/Mugiliformes) |  |  |  |
|  | [*Mugilidae*](https://en.wikipedia.org/wiki/Mugilidae) (Mullets) |  |  |
|  |  | *Mugil* | *M. cephal* (Flathead grey mullet) |
|  |  | *Liza* | *L.* sp. |
| [*Beryciformes*](https://en.wikipedia.org/wiki/Beryciformes) |  |  |  |
|  | [*Holocentridae*](https://en.wikipedia.org/wiki/Holocentridae) |  |  |
|  |  | *Myripristis* | *M. murdjan* (Blotcheye soldierfish) |
| [*Pleuronectiformes*](https://en.wikipedia.org/wiki/Pleuronectiformes) |  |  |  |
|  | [*Scophthalmidae*](https://en.wikipedia.org/wiki/Scophthalmidae) |  |  |
|  |  | *Scophthalmus* | *S. maximus* (Turbot fish) |
| [*Osmeriformes*](https://en.wikipedia.org/wiki/Osmeriformes) |  |  |  |
|  | *Plecoglossidae* |  |  |
|  |  | *Plecoglossus* | *P. altivelis* (Ayu fish) |
| [*Siluriformes*](https://en.wikipedia.org/wiki/Siluriformes) |  |  |  |
|  | [*Pangasiidae*](https://en.wikipedia.org/wiki/Pangasiidae) |  |  |
|  |  | *Pangasius* | *P. hypophthalmus* (Tra fish) |
|  | [*Doradidae*](https://en.wikipedia.org/wiki/Doradidae) |  |  |
|  |  | [*Platydoras*](https://en.wikipedia.org/wiki/Platydoras) | *P. costatus* (Raphael catfish) |
|  | *Ariidae* |  |  |
|  |  | [*Ariopsis*](https://en.wikipedia.org/wiki/Ariopsis_%28fish_genus%29) | *A. felis* (Hardhead sea catfish) |
| *Characiformes* |  |  |  |
|  | [*Characidae*](https://en.wikipedia.org/wiki/Characidae) |  |  |
|  |  | *Paracheirodon* | *P. axelrodi* (Cardinal tetra) |
| *Clupeiformes* |  |  |  |
|  | *Engraulidae* |  |  |
|  |  | *Stolephorus* | *S.* sp. |
